# Application progress of CRISPR/Cas9 genome-editing technology in edible fungi

**DOI:** 10.3389/fmicb.2023.1169884

**Published:** 2023-05-25

**Authors:** Yan Zhang, Shutong Chen, Long Yang, Qiang Zhang

**Affiliations:** College of Plant Protection, Shandong Agricultural University, Tai'an, China

**Keywords:** edible fungi, CRISPR/Cas9, molecular breeding, application progress, genome editing

## Abstract

Edible fungi are not only delicious but are also rich in nutritional and medicinal value, which is highly sought after by consumers. As the edible fungi industry continues to rapidly advance worldwide, particularly in China, the cultivation of superior and innovative edible fungi strains has become increasingly pivotal. Nevertheless, conventional breeding techniques for edible fungi can be arduous and time-consuming. CRISPR/Cas9 (clustered regularly interspaced short palindromic repeats/CRISPR-associated nuclease 9) is a powerful tool for molecular breeding due to its ability to mediate high-efficiency and high-precision genome modification, which has been successfully applied to many kinds of edible fungi. In this review, we briefly summarized the working mechanism of the CRISPR/Cas9 system and highlighted the application progress of CRISPR/Cas9-mediated genome-editing technology in edible fungi, including *Agaricus bisporus, Ganoderma lucidum, Flammulina filiformis, Ustilago maydis, Pleurotus eryngii, Pleurotus ostreatus, Coprinopsis cinerea, Schizophyllum commune, Cordyceps militaris*, and *Shiraia bambusicola*. Additionally, we discussed the limitations and challenges encountered using CRISPR/Cas9 technology in edible fungi and provided potential solutions. Finally, the applications of CRISPR/Cas9 system for molecular breeding of edible fungi in the future are explored.

## 1. Introduction

Edible fungi have long been valued worldwide for their rich nutritional content, medicinal benefits, and pleasant taste (Pérez et al., 2021). With the rapid expansion of the edible fungi market, the need for high-quality and high-yield strains has been rapidly increasing. At present, there are various defects in the existing edible fungi production, such as strain degeneration (Zhu et al., 2020; Pérez et al., 2021; Chen et al., 2022b), unstable quality, poor resistance to diseases and insects, and easily becoming soft and brown during storage (Zhang et al., 2021). However, the traditional breeding method is inefficient, time-consuming, and labor-intensive (Boontawon et al., 2021b), and the breeding direction is uncertain, which limited molecular breeding for the improvement of cultivars. Consequently, molecular breeding techniques, such as genome editing, have emerged as a promising alternative.

Genome editing is a novel technology for manipulating genomic DNA sequences at specific sites (Osakabe et al., 2016; Tehrani et al., 2019). Zinc-finger nucleases (ZFNs) and transcription activator-like effector nucleases (TALENs) were the first generation of genome-editing techniques but were complex, inefficient, and costly (Hsu et al., 2014; Juillerat et al., 2014), which limited their widespread application in edible fungi. In contrast, the CRISPR/Cas9 system is simple, flexible, stable, and cost-effective, making it a more practical option for genome editing in edible fungi (Xiao-tian et al., 2021). In this review, we provided a comprehensive summary of recent applications of CRISPR/Cas9 in the genetic improvement of edible fungi. We also highlighted potential issues and future prospects of this technology in the field of molecular breeding. Overall, our review aimed to provide a serviceable reference for researchers and practitioners interested in the molecular breeding of edible fungi.

## 2. Features of the application of CRISPR/Cas9 in edible fungi

**Figure 1:**
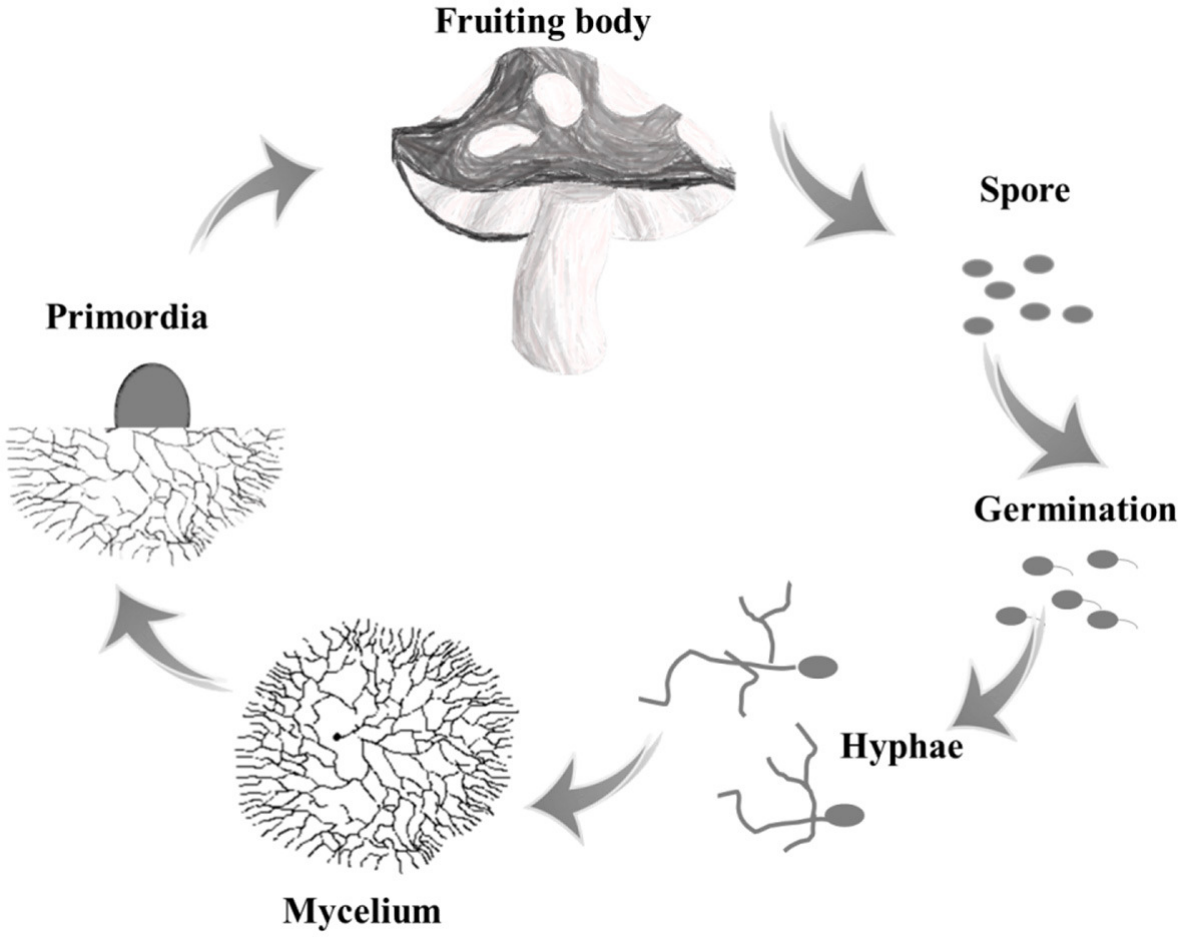
The typical lifecycle of edible fungi.

Edible fungi are a group of macro-fungus that can form large fleshy or colloidal fruit bodies or sclerotia tissues, which mainly included basidiomycetes and ascomycetes. Taking basidiomycetes as an example, the lifecycle of a mushroom begins with the spores, which are released from the basidium and dispersed by wind. When the spores land in a suitable environment, they germinate and grow into threadlike structures called hyphae. These hyphae then fuse together to form mycelium, which is the vegetative part of the fungus that grows underground or within a substrate. As the mycelium matures, it produces fruiting bodies, which are the mushrooms that we see above ground (Figure 1) (Gupta et al., 2018). The initial stage of a sub-entity that lacks organizational differentiation in appearance is usually referred to as a primordium or abbreviated as primordia.

Edible fungi are rich in nutrients, such as protein, fiber, vitamins, and minerals, and have been found to have anti-inflammatory and immune-boosting properties. In recent years, CRISPR/Cas9 genome-editing technology was applied in the molecular breeding of edible fungi to improve the yield and quality of edible fungi. The working principle of the CRISPR/Cas9 system has been explained in detail in many studies (Jinek et al., 2012; Barrangou and Marraffini, 2014; Sander and Joung, 2014; Wang and Coleman, 2019; Ullah et al., 2020), which will not be elaborated on here. The CRISPR/Cas9 system works in edible fungi similar to the manner described in the abovementioned literature but with specific application features. CRISPR-related research in edible fungi is relatively lagging behind (Xiao-tian et al., 2021) compared with that in plants and mammals, due to the fact that most edible fungi are not model fungi in the traditional sense, coupled with complex genetic background and immature genetic manipulation methods.

**Figure 2:**
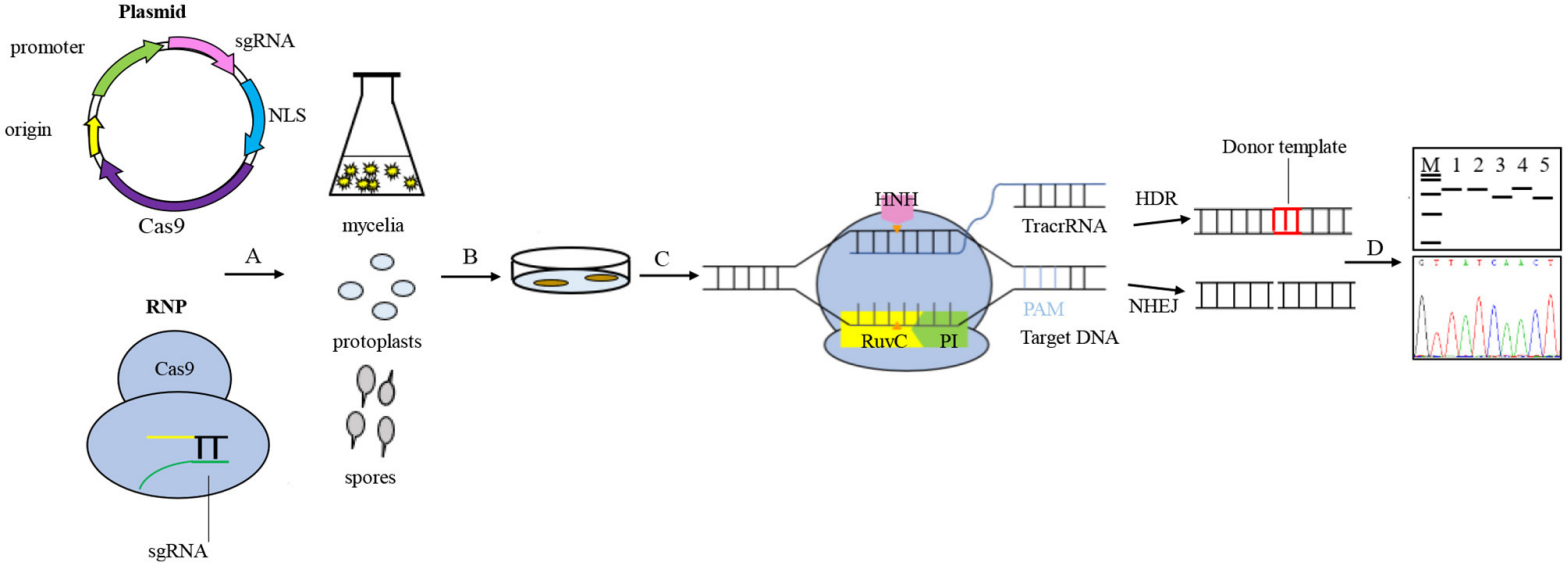
General procedures for genome editing in edible fungi using the CRISPR/Cas9 system. Vector construction generally connects the *Cas9* gene and sgRNA to a single vector or a double vector, or using pre-assembled Cas9-RNPs. A. The vectors were transported to target cells and integrated into the target genome through either polyethylene glycol (PEG) transformation or *Agrobacterium*-mediated transformation (AMT). The host receptor materials for edible fungi transformation include protoplasts, spores, and mycelia. B. The transformants were screened based on the traits shown by the screening marker genes. C. The process of genome editing involves the Cas9 enzyme specifically recognizing and binding to genomic target sites, guided by sgRNA. The HNH and RuvC nuclease active domains of the Cas9 protein are responsible for cleaving 3- to 4-bp upstream of the protospacer adjacent motif (PAM) site of the target DNA, resulting in double-strand breaks (DSBs) and stimulating the genomic DNA to initiate a self-repair mechanism. The homologous recombination (HR) pathway precisely edits the target gene guided by the donor DNA fragment, while the non-homologous end joining (NHEJ)-dominated repair pathway ligates the break ends directly without using a homologous template, which can sometimes cause targeted mutations, such as random loss, insertion, or base substitutions. D. The target gene mutants are screened and verified using diagnostic PCR and Sanger sequencing.

In the CRISPR system vector of edible fungi, constitutive promoters, such as t*rpC, gpdA*, and *tef1* promoters from *Aspergillus nidulan* (Svahn et al., 2015; Zhang et al., 2016; Song et al., 2019), have been employed to activate the expression of the Cas9 protein. The sgRNA typically undergoes transcription *in vitro* driven by the *T7* promoter and *in vivo* driven by the *U6* promoter (Gao and Zhao, 2014; Shi et al., 2017). The typical CRISPR/Cas9 system vector for fungal genome editing involves the connection of the *Cas9* gene and sgRNA to either a single vector or a double vector. The complex is then delivered to the target cells and integrated into the target genome via either polyethylene glycol (PEG) transformation or Agrobacterium-mediated transformation (AMT) (Song et al., 2019). Furthermore, a few studies have successfully achieved transformants of edible fungi using pre-assembled Cas9-RNPs (Cas9-guide RNA ribonucleoproteins). There are a variety of options available for the transformation of host receptor materials in edible fungi due to their unique characteristics. These options include protoplasts, spores, or mycelia. However, the selection of screening markers in edible fungi is limited. Available markers include genes that mediate drug resistance, autotrophic markers, nutritional genes, and visual distinction reporters (Deng et al., 2017a), such as carboxin and hygromycin resistance genes (*hph*) and uracil nutrient deficiency markers. The use of CRISPR/Cas9 technology for molecular breeding of edible fungi is not only more convenient but also more efficient than traditional breeding methods (Figure 2). Despite the late adoption of the CRISPR/Cas9 technology in the field of edible fungi, a significant number of studies have been conducted in recent years. Here, we highlight the progress of the CRISPR/Cas9 system in the application of edible fungi (Table 1).

**Table 1:** Applications of CRISPR/Cas9 in edible fungi.

**Species**	**Promoter for gRNA**	**Promoter for Cas9**	**Target gene**	**Selective marker**	**Materials**	**Methods**	**Transformation efficiency**	**Editing efficiency**	**References**
*Agaricus bisporus*	*U6*	*gpd*	*ppo4*	*hyg^*r*^*	Mycelia	AMT	—	16.67	2020 (Qin et al., 2017)
*Ganoderma lucidum*	*T7 in vitro* transcription	*gpd*	*ura3*	*cbx^*r*^*	Protoplast	PMT	66.67	0.2–1.78/10^7^ protoplasts	2017 (Wang P. A. et al., 2022)
*Ganoderma lucidum*	*T7 in vitro* transcription	*gpd*	*ura3/GL17624*	*bar^*r*^*	Protoplast	PMT	—	36.7/13.33	2020 (Luo et al., 2016)
*Ganoderma lucidum*	*U6*	*gpd*	*ura3/cyp5150l8/cyp505d13*	*cbx^*r*^*	Protoplast	PMT	—	5.3/0.67/2/10^7^ protoplasts	2020 (Wang et al., 2018)
*Ganoderma lucidum*	*U6*	*Pgpd*	*glcrz1/glcrz2*	*cbx^*r*^*	Protoplast	PMT	—	3.57/17.86	2020 (Tu et al., 2021)
*Ganoderma lucidum*	*U6*	*Pgpd*	*ura3/ku70*	*hyg^*r*^*	Protoplast	PMT	—/60/10^7^ protoplasts	93.1(Replacement), 96.3(Insertion)/–	2021 (Ouyang et al., 2018)
*Ganoderma lucidum*	*U6*	*Pgpd*	*glcrz2/cyp5150l8*	*hyg^*r*^*	Protoplast	PMT	—	12.5/12.5	2022 (Sakamoto et al., 2004)
*Flammulina filiformis*	*H1*	*gpd-fvs*	*Mads-8*	*hyg^*r*^*	Protoplast	PMT	—	11.11	2016 (Liu et al., 2017)
*Flammulina filiformis*	none	*gpd*	*none*	*hyg^*r*^*	Mycelia	AMT	6.84	—	2017 (Kraft et al., 2015)
*Flammulina filiformis*	*H1*	*gpd*	*HK1/HK2*	*hyg^*r*^*	Protoplast	PMT	24.1/12.5	—	2018 (Liu J. et al., 2022)
*Flammulina filiformis*	*gpd*	*gpd*	*Fvgpcr1/Fvgpcr2*	*hyg^*r*^*	Mycelia	AMT	75	0	2019 (Wang Y. et al., 2022)
*Flammulina filiformis*	*U6*	*gpd*	*pyrG*	5-FOA	Protoplast	PMT	—	16.67	2022 (Wege et al., 2021)
*Flammulina filiformis*	synthesis *in vitro*	synthesis *in vitro*	*pyrG*	5-FOA	Protoplast	PMT	5/10^7^ cells mL^−1^	100	2022 (Liu X. et al., 2022)
*Ustilago maydis*	*U6*	*Otef*	*bE/bW*	*cbx^*r*^*	Protoplast	PMT	90–140	70	2016 (Khanal et al., 2021)
*Ustilago maydis*	*U6*	*Otef*	*um03848/um03849*	*hyg^*r*/^cbx^*r*^*	Protoplast	PMT	—	—	2021 (Sugano et al., 2017)
*Ustilago maydis*	*U6*	*Otef*	*don3/rua1/emt1/mat1/cdk5*	*cbx^*r*^*	Protoplast	PMT	—	50/—/—/—/16.67	2021 (Koshi et al., 2022)
*Pleurotus eryngii*	*U6*	*gpd*	*pyrG*	*cbx^*r*^*	Protoplast	PMT	—	78.9	2021 (Jan Vonk et al., 2019)
*Pleurotus ostreatus*	*U6*	*ef3*	*pyrG/fcy1*	*hyg^*r*^*	Protoplast	PMT	47.1/78.6	20–94.9	2021 (Tehrani et al., 2019)
*Pleurotus ostreatus*	Synthesis *in vitro*	*synthesis in vitro*	*pyrG*	*hyg^*r*^*	Protoplast	PMT	100	7–100	2021 (Suhadolnik and Cory, 1964)
*Pleurotus ostreatus*	*U6*	*ef3*	*pcc1/clp1*	*hyg^*r*^*	Protoplast	PMT	—	60/60	2021 (Reis et al., 2013)
*Pleurotus ostreatus*	*U6*	*ef3*	*mer3/msh4*	*hyg^*r*^*	Protoplast	PMT	—	21.05	2022 (Das et al., 2010)
*Pleurotus ostreatus*	*U6*	*ef3*	*fcy1*	*hyg^*r*^*	Protoplast	PMT	6.52	—	2022 (Taofiq et al., 2016)
*Coprinopsis cinerea*	*U6*	*CcDED1*	*gfp*	*hyg^*r*^*	Protoplast	PMT	7–28/10^8^ cells	10.5	2017 (Chen et al., 2018)
*Schizophyllum commune*	*In vitro* transcription	*heterologous expression*	*hom2*	*nour^*r*^*	Protoplast	PMT	—	0.15–1.8/10^7^ protoplasts	2019 (Chen et al., 2022a)
*Cordyceps militaris*	*T7 in vitro* transcription	*gpd*	*ura3*	*blpR^*r*^*	Conidium	AMT	64.71	11.76	2018 (Li et al., 2017)
*Cordyceps militaris*	*PtrpC*	*Pgpd*	*ura3*	5-FOA	Protoplast	PMT	—	30.56	2022 (Foster et al., 2018)
*Cordyceps militaris*	*tRNA_*Pro*_/tRNA_*Glu*_/ AfU6-tRNA_*Gly*_*	*Pgpd*	*Cmwc-1/Cmvvd*	*hyg^*r*^*	Protoplast	PMT/AMT	92.11/10	55.1/89/73.9/10	2022 (Liu W. et al., 2020)
*Shiraia bambusicola*	*U6*	*ef1α*	*SbaPKS*	*hyg^*r*^*	Protoplast	PMT	—	32	2017 (Liu et al., 2015)
*Shiraia bambusicola*	*U6-1/U6-2/U6-3/5SrRNA*	*Pgpd*	*pyrG/ku80*	5-FOA	Protoplast	PMT	—	100	2020 (Shi et al., 2019)

PMT, PEG-mediated transformation; AMT, *Agrobacterium*-mediated transformation; “—” indicates that the data were not reported.

To successfully apply CRISPR/Cas9 to edible fungi, several key requirements must be met: (1) Selecting the appropriate CRISPR/Cas9 system: The CRISPR/Cas9 system must be carefully selected based on the type of edible fungus being studied. The system must be efficient, accurate, and specific to ensure successful gene editing. (2) Design of sgRNA: The sgRNA must be designed to target specific genes of interest. They must also be specific enough to avoid off-target effects, which could lead to unintended consequences. (3) Transformation methods: The CRISPR/Cas9 components must be delivered into fungal cells using an appropriate transformation method. This can include protoplast transformation or Agrobacterium-mediated transformation. (4) Validation of gene editing: Gene editing must be validated to ensure that the desired changes have been made. This can be accomplished through PCR analysis, sequencing, or other molecular techniques. (5) Optimization of growth conditions: The growth conditions of edited fungi must be optimized to ensure proper growth and development. This may include factors, such as temperature, light, and nutrient availability.

Overall, the successful application of CRISPR/Cas9 in edible fungi requires careful selection of the CRISPR/Cas9 system, design of guide RNA, transformation method, validation of gene editing, and optimization of growth conditions. These requirements pave the way for using gene editing to enhance the yield and quality of edible fungi.

## 3. Application progress of CRISPR/Cas9 in edible fungi

Recent advancements in molecular biology, genomics, and bioinformatics, coupled with the relentless efforts of scientific researchers, have led to the widespread application of the CRISPR/Cas9 genome-editing system in many edible fungi, including *Agaricus bisporus, Flammulina filiformis, Pleurotus eryngii, Pleurotus ostreatus, Cordyceps militaris*, and so on.

### 3.1. Applications of CRISPR/Cas9 genome-editing technology in *Agaricus bisporus*

*Agaricus bisporus*, which belongs to the basidiomycota class, is one of the most extensively cultivated and consumed edible fungi in the world (Cai et al., 2022). Browning in *A. bisporus* caused by polyphenol oxidase (*PPO*) is a common and economically detrimental phenomenon, which inevitably influences the product quality, sensory acceptance, and nutritional value of the mushroom, resulting in certain economic losses (Sun et al., 2012; Wang et al., 2017). Various genetic manipulation experiments have been applied in the cultivation of anti-browning *A*. *bisporus*. In 2016, one of six *PPO* genes was knocked out by CRISPR/Cas9 in the *A*. *bisporus*, reducing the enzyme's activity by 30% and showing the ability to resist browning. It is the first successful application of the CRISPR/Cas9 system in an edible fungi (Waltz, 2016). The edited button mushroom received a green light from the United States Department of Agriculture (USDA), as it had not transferred foreign genes. The research marks a technical milestone. Similar research was conducted by Yang et al. (2020). *AbPPO4* is a browning-related gene, which is highly expressed in fruiting bodies (Wu et al., 2010). To further clarify the browning mechanism of *A. bisporus*, the genome-editing vector pAbGEB1-AbPPO4 carrying the *hph* was successfully constructed and then transformed into *A*. *bisporus* mycelium cells by *Agrobacterium*-mediated transformation (Yang et al., 2020). The results showed that the designed CRISPR/Cas9 system successfully edited the *AbPPO4* gene in *A. bisporus* and it was also found that the mutation of *AbPPO4* gene had an adverse effect on the growth of its mycelium. In conclusion, the *AbPPO4* gene is likely to not only participate in the browning-related phenotype of *A. bisporus*, but also play an important role in the growth and development of *A. bisporus*.

### 3.2. Applications of CRISPR/Cas9 genome-editing technology in *Ganoderma lucidum*

*Ganoderma lucidum* is an esteemed traditional Chinese medicinal material that is known for its health-boosting properties. It can synthesize a variety of biologically active substances with anti-tumor and anti-bacterial effects (Zhong and Xiao, 2009; Xiao and Zhong, 2016), such as highly oxygenated lanostane-type triterpenoids–ganoderic acids (GAs). However, the synthesis of GAs in *G. lucidum* is hindered by the orotidine 5′-monophosphate decarboxylase gene (*ura3*) (Pei et al., 2018). In 2017, Qin et al. (2017) established the CRISPR/Cas9 genome-editing system in both *G. lucidum* and *Ganoderma lingzhi* for the first time and further promoted *ura3* gene disruption. First, the CRISPR/Cas9 expression vector pMD18-Glcas9 carrying the carboxin resistance gene was constructed, which was driven by the *Pgpd* promoter and then transformed into the protoplasts of *G. lucidum* using a modified PEG-mediated transformation method. The *G. lucidum* strain Gl-Cas9 capable of expressing Cas9 protein was successfully obtained with a conversion efficiency of 66.6%. The sgRNAs transcribed *in vitro* and driven by the *T7* promoter were transferred into the *G. lucidum* strain that successfully expressed Cas9 protein. The two designed sgRNAs targeting different sites of *ura3* finally yielded 3 and 5 uracil nutrition-deficient strains. The researchers hoped to improve the editing efficiency by a one-time transformation of sgRNA and Cas9 gene expression elements but unfortunately failed in the end. Despite this setback, this study accelerated strain improvement through rational genetic methods, helping to provide a broadly applicable gene-knockout methodology in higher fungi.

Previous studies demonstrated that adding calcium ions during *G. lucidum* mycelial fermentation could significantly increase the yield of GA (Xu and Zhong, 2012). Calcineurin-responsive zinc finger transcription factor CRZ1 is a key transcription factor for calcium signal transduction. In 2020, Li and Zhong (2020) utilized a bioinformatic analysis to identify two proteins, GlCRZ1 and GlCRZ2, in the *G. lucidum* proteome that displayed the highest similarity to CRZ1. The *crz1* homologous genes *glcrz1* and *glcrz2* were individually knocked out using the CRISPR/Cas9 genome-editing system with carboxin resistance and PEG-mediated protoplast transformation methodology. The mutants and wild-type strains were cultured under different calcium ion conditions, including the absence of calcium ions, a high concentration of calcium ions (100 mM), and a low concentration of calcium ions (10 mM), to observe the growth of mycelia and the yield of GA. Compared with the wild-type strain, the addition of Ca^2+^ enhanced GA production, and the disruption of *glcrz1* did not impede cell growth, but abolished the effect of calcium signal on stimulating GA biosynthesis. For *glcrz2*-knockout strains, even with Ca^2+^ treatment, their biomass and GA production were hampered severely. These results indicated that *glcrz1* was required to transmit calcium signals to regulate GA synthesis, and *glcrz2* was essential for cell growth and GA secondary metabolism. This study provides valuable insights into the role of CRZ1 in regulating secondary metabolism in basidiomycetes mushrooms. In 2020, Wang et al. (2020) constructed the CRISPR/Cas9 genome-editing system in *G. lucidum* to study the functional genes involved in GA biosynthesis. The researchers evaluated the efficiency of *ura3* disruption using various sgRNA constructs containing an endogenous promoter and self-cleaving ribozyme HDV. They also identified a *U6* promoter suitable for *in vivo* expression of sgRNA. Then, the *ura3* disruption plasmid was constructed and transformed into the protoplasts of *G. lucidum* by PEG-mediated transformation. The highlight is that HDV ribozymes were introduced at the 3′ end of the sgRNA to achieve precise transcriptional termination to improve gene-editing efficiency. This newly developed system was subsequently applied to precisely edit the functional gene *cyp5150l8*, which is responsible for transforming lanosterol to GA. Compared with the wild-type strain, the titers of the four identified GAs in the *cyp5150l8* disruptant were significantly reduced. To test the editing efficiency of this system in targeting other functional genes in *G. lucidum*, another *cyp505d13* gene involved in the bioproduction of squalene-type triterpenoid 2,3; 22,23-squalene dioxide was also successfully disrupted with an editing efficiency of 22.2%. This study is of great significance for strain molecular breeding and metabolic engineering of *G. lucidum* and other basidiomycetes mushrooms.

To further clarify the biosynthetic pathway of metabolites in *G. lucidum*, Wang P. A. et al. (2022) applied CRISPR/Cas9-assisted *in situ* complementation in *G. lucidum*, which is the first report on the application of CRISPR/Cas9 to complement mutants *in situ* in basidiomycetes. The results showed that the content of four GAs was restored in the *in situ* complemented strains of *glcrz2* and *cyp5150l8*. This study is of great value for the identification of various genes of other basidiomycetes. In 2020, Liu K. et al. (2020) increased the frequency of CRISPR/Cas9-mediated gene disruption in *G. lucidum* by adding an intron upstream of the *Cas9* gene, which is indispensable for the efficient expression of heterologous phosphinothricin-resistance gene (*bar*) and green fluorescent protein gene (*gfp*) in *G. lucidum*. The four sgRNA cassettes driven by *T7* promoter, including two *ura3* and two *GL26016* gene targeting sequences, were transformed into the protoplasts of *G. lucidum* by PEG-mediated genetic transformation, and then, the transformants were screened on specific selection plates. The dual sgRNA-mediated CRISPR/Cas9 system established in this study successfully achieved the deletion of *ura3* and *GL17624* genes in *G. lucidum*. The deletion frequency was significantly improved, making this system a valuable tool for creating gene deletion mutants and studying gene function in *G. lucidum* and other basidiomycetes. In 2021, Tu et al. (2021) no longer focused on the knockout of the target gene *ura3* in *G. lucidum*, but broadened their horizons to study the insertion and replacement of *ura3*, which remains a serious challenge due to the low efficiency of homologous recombination in *G. lucidum*. To eliminate the interference of the main repair pathway of DSBs induced by the Cas9-NHEJ repair pathway, the sgRNA-directed gene deletion method was first used to inactivate the NHEJ repair mechanism by disrupting the Ku70 gene (*Ku70*) using the CRISPR/Cas9 system. The plasmid carrying the codon-optimized *hph* was constructed and introduced into *G. lucidum* protoplasts by PEG-mediated transformation, and *ku70* mutants were successfully obtained. When the target DNA with *ura3* gene and 1.5-kb homologous 5′- and 3′-flanking sequences were used as donor templates, the insertion and replacement frequencies of the target gene in *ku70* mutant were 96.3 and 93.1%, respectively, while those in the control strain (Cas9) were 3.3 and 0%, respectively. The results demonstrated that the *ku70* mutant was an effective receptor for gene insertion and replacement. This study provided a reference for the targeted insertion and replacement of genes in *G. lucidum*, which will promote our in-depth study of the gene function of *G. lucidum*.

### 3.3. Applications of CRISPR/Cas9 genome-editing technology in *Flammulina filiformis*

*Flammulina filiformis*, previously referred to as *F. velutipes*, is a globally important edible basidiomycetes for its nutritional value and medicinal properties worldwide (Wang et al., 2018). However, the hyphae of *F. velutipes* must be stimulated at low temperatures (10–15°C) to promote the formation of primordium and further develop into fruit bodies (Sakamoto et al., 2004), which leads to some problems in the large-scale production, such as high energy consumption, high cost, and heavy pollution. Therefore, it is of great scientific significance to explore the growth and development mechanism of *F. velutipes* fruit body by molecular biology methods.

In 2016, Luo et al. (2016) first explored the editing effect of a CRISPR/Cas9 editing system on *F. velutipes* genome. Based on the gene transcriptome data of *F. velutipes* mycelium and primordium stages, the knockout vector pgFvs-cas9-sgRNA of *Mads-8* gene related to growth and development was successfully constructed, and the vector was transformed into protoplast using the PEG-mediated method. In 2018, Ouyang et al. (2018) constructed a CRISPR/Cas9 system of *F. velutipes* that targets genes involved in cold-induced fruiting body formation. The researchers established CRISPR/Cas9 vectors pgfvs-Cas9-HK1-sgRNA and pgfvs-Cas9-HK2-sgRNA for cold-inducible genes *HK* 1 and *HK* 2, respectively, which are related to primordium formation in *F. velutipes*. These vectors were transformed into *F. velutipes* by PEG-mediated protoplast transformation with transformation rates of 24.1 and 12.5%, respectively. The abovementioned studies showed that the recombinant vector was successfully transformed into the genome of *F. velutipes*, providing a theoretical foundation for further exploring the growth mechanism of *F. velutipes* and cultivating high-quality *F. velutipes* strains.

In 2019, Lin et al. (2019) reported a study on the knockout of functional genes in *F. velutipes* using the CRISPR/Cas9 system. Two putative G protein-coupled receptor genes *Fvgpcr1* and *Fvgpcr2* in *F. velutipes* were obtained by amino acid homology alignment (Blast P) and transcriptome data (Cabrera et al., 2015). To target these genes, four expression vectors were constructed: pCAMBIA0390-hph-Fvcas9-Fvgpcr1-sgRNA1/sgRNA2; pCAMBIA0390-hph-Fvcas9-Fvgpcr2-sgRNA1/sgRNA2. The vectors were successfully integrated into the genome of *F. velutipes* by *Agrobacterium*-mediated transformation of *F. velutipes* mycelium. Unfortunately, the FvCas9 protein was normally expressed, but no *Fvgpcr* gene-knockout mutant was obtained. In 2017, Lin et al. optimized the Streptococcus pyogenes Cas9 (SpCas9) codon to synthesize the full-length sequence of FvCas9 and constructed the binary expression vector FpiC. The mycelia of *F. velutipes* monokaryon “Dan3” transformed by the *Agrobacterium*-mediated method were used to obtain transformants. However, the transformation rate was low, only 6.84% (Liu et al., 2017); 5 years later, Liu J. et al. (2022) first established a CRISPR/Cas9 genome-editing system in the mushroom *F. velutipes* using a fast, accurate, and transgene-free RNP complex delivery technology (Wang P. A. et al., 2022), greatly improving the targeting efficiency to 100%. In this study, a CRISPR/Cas9 genome-editing method based on *in vitro* assembled Cas9 and RNPs was developed, which avoids the use of any exogenous DNA, thus saving time and manpower in plasmid construction.

The dual sgRNA-mediated fragment deletion strategy has attracted increasing attention (Zhou et al., 2014; Kraft et al., 2015), possibly due to its high efficiency and divergent visualization (Liu X. et al., 2022). In 2022, Liu X. et al. (2022) successfully established a CRISPR/Cas9 genome-editing system based on double sgRNAs in *F. velutipes* for the first time. Under the control of the *gpd* promoter and *FfU6* promoter, plasmids harboring basidiomycetes codon-optimized Cas9 expression cassette and dual sgRNAs targeting *pyrG* (orotidine 5′ phosphate decarboxylase gene) were transferred into the protoplasts of the *F. velutipes* strain Dan3 by PEG-mediated transformation. Finally, several *pyrG* mutants with fragment deletions or small insertions and deletions (indels) were identified, resulting in genetic alterations in genomic information. This study showed a good example of the application of advanced tools in cultivated mushrooms.

### 3.4. Applications of CRISPR/Cas9 genome-editing technology in *Ustilago maydis*

*Ustilago maydis* is a kind of basidiomycetes; often eating it can not only protect the liver system but also help digestion and defecation, and is the pathogen of corn smut disease as well. In 2016, Schuster et al. (2016) reported the establishment of the CRISPR/Cas9 system for generating targeted alterations in the genome of the *U. maydis*. *bE* and *bW* form an active homeodomain complex in non-allelic combinations triggering filamentation and pathogenic development (Kämper et al., 1995). Due to the presence of an active bE-bW complex and autocrine pheromone stimulation, these strains can form filamentous colonies (Fuz^+^ phenotype) on solid media containing charcoal (Banuett and Herskowitz, 1989). Thus, *bE* and *bW* are used as marker genes to screen mutants in *U. maydis*. The plasmids pCas9_sgRNA_bW2 and pCas9_sgRNA_bE1 were generated and introduced into the *U. maydis* SG200 strain by PEG-mediated protoplast transformation. Colony screening and sequencing showed that the mutation efficiency was up to 70%. This technology greatly improves the genetic efficiency of *U. maydis*. In 2021, Wege et al. (2021) created several different types of mutants in *U. maydis* using the CRISPR/Cas9 technology. These include targeted gene deletion through homologous recombination of short double-stranded oligonucleotides, introduction of point mutations, heterologous complementation at genomic sites, and endogenous N-terminal labeling using the fluorescent protein mCherry. All applications do not rely on permanent selection markers, and only transient expression of endonuclease Cas9hf and sgRNA is required. The techniques demonstrated in this study have the potential to accelerate the study of maize smut communities but could also serve as a template for genome editing in other important fungi.

*U. maydis* can use nitrate as the sole nitrogen source. Khanal et al. (2021) characterized the role of nitrate assimilation genes in the virulence of *U. maydis*, including two genes, *um03848* and *um03849*, which serve as nitrite reductase and nitrate transporter, respectively. The deletion mutants for *um03848, um03849*, or both genes were obtained by homologous recombination. Additionally, the pCas9-um03849 plasmid carrying carboxin resistance gene was constructed and used to transform protoplasts of compatible *U. maydis* mating strains to produce INDEL mutation. All mutants obtained by CRISPR-mediated and homologous recombination were examined, and it was inferred that genes *um03849* and *um03848* were used for nitrate utilization alone or together. This is the first study to verify the role of nitrate assimilation genes in maize and opens up a possible avenue for further study of nitrogen assimilation pathways using *U. maydis* as a model organism.

### 3.5. Applications of CRISPR/Cas9 genome-editing technology in *Pleurotus eryngii*

*Pleurotus eryngii*, classified as basidiomycetes, has been widely cultivated in many countries, especially in China (Wang et al., 2021). *P. eryngii* contains a plethora of unique and versatile bioactive compounds. Nevertheless, studies on functional genes involved in the synthesis of these compounds have been severely impeded by the absence of a mature and effective gene-editing system for *P. eryngii*. In 2021, Wang et al. (2021) innovatively established the CRISPR/Cas9 system in *P. eryngii* and improved the genetic transformation system. At first, they determined that the endogenous *sdhB* gene point-mutated variant (*cbx*^*r*^) was a more stable selective marker than *hph*. In addition, the codon of the Cas9 protein was further optimized, and a native efficient *U6* promoter was selected to guide sgRNA transcription, thereby improving the genome-editing efficiency. A more efficient and stable *pyrG* gene-editing system was established in *P. eryngii* by optimizing the PEG-mediated protoplast transformation system. Different insertions and deletions were incorporated through NHEJ and HDR to realize the genome-editing function of the *pyrG* system. Although this system has not yet been used for gene editing of functional genes in *P. eryngii*, it is just around the corner based on the establishment of the CRISPR/Cas9 system of this study. This study paves the way for gene function research and molecular breeding of *P. eryngii*.

### 3.6. Applications of CRISPR/Cas9 genome-editing technology in *Pleurotus ostreatus*

*Pleurotus ostreatus* is one of the most economically important mushroom species and possesses both edible and medicinal value (Boontawon et al., 2021c). Extensive breeding of *P. ostreatus* has improved its cultivation and food qualities (Sonnenberg et al., 2017; Barh et al., 2019). However, these classical breeding methods are laborious and time-consuming. Thus, methodologies for more time- and cost-effective molecular breeding are needed (Yamasaki et al., 2022). The implementation of the CRISPR/Cas9 system in *P. ostreatus* will undoubtedly facilitate its molecular breeding. An efficient genome-editing system for non-genetically modified (non-GM) in *P. ostreatus* using plasmid-based CRISPR/Cas9 was constructed (Boontawon et al., 2021b). Plasmids harboring Cas9 expression cassettes and different sgRNAs targeting *fcy1* and *pyrG* were individually transferred into protoplasts of the PC9 strain. Mutants with resistance to 5-fluorocytosine and 5-fluoroorotic acid (5-FOA) were obtained. Small insertions/deletions or insertion of plasmid fragments at the target site were found in some resistant strains, which proved that CRISPR/Cas9-assisted genome editing of *P. ostreatus* is available. It is the first step toward using CRISPR/Cas9 for modern molecular breeding of non-GM cultivated strains in the future. Soon after, Boontawon et al. (2021c) realized plasmid-free genome editing in *P. ostreatus* using Cas9 RNP, and strains resistant to 5-FOA were generated using this method. The results demonstrated Cas9 RNP-assisted gene mutation could be used for the molecular breeding of *P. ostreatus* as well as other edible fungi.

The formation of dikaryons in *Agaricomycetes* is mainly controlled by A and B mating-type loci. Based on the results of previous studies, Boontawon et al. (2021a) speculated the molecular mechanism of dikaryon formation was related to *pcc1* and *clp1*. Such plasmids harboring the Cas9 expression cassette and different sgRNAs targeting *pcc1* or *clp1* were introduced into strain PC9 to obtain mutants. The dikaryosis of the *clp1* mutant was damaged, suggesting that the function of *clp1* may be necessary for clamp cell formation. This study not only provides a successful example of the application of CRISPR/Cas9 in functional studies of cultivated mushrooms but also provides a reference for further revealing the molecular mechanism of sexual development in *Agaricomycetes*. In addition to the monokaryotic *P. ostreatus* strain PC9, CRISPR/Cas9 has also been successfully performed on the dikaryotic *P. ostreatus*. In 2022, Yamasaki et al. (2022) reported a plasmid-based CRISPR/Cas9 technique for transforming both nuclei of dikaryotic *P. ostreatus*, PC9 × #64, to generate strains with low basidiospore production ability. Meiosis-related genes *mer3* or *msh4* were selected as target genes to design two different sgRNAs. These four different plasmids were introduced into PC9 × # 64 to obtain mutants with low basidiospore production ability, so as to develop an efficient and rapid molecular breeding method for cultivated mushrooms. Koshi et al. (2022) achieved marker-free genome editing for the first time by transiently expressing Cas9, sgRNA, and *hph* in *P. ostreatus*. The gene of *fcy1* was edited by transiently expressing Cas9, sgRNA, and *hph*, and strains with 5-fluorocytosine resistance and hyg sensitivity were isolated. The genome-editing effect of *fcy1* in the mutant strains was confirmed through Sanger sequencing. This significant achievement marks a new chapter in fungal repetitive genome editing.

### 3.7. Applications of CRISPR/Cas9 genome-editing technology in *Coprinopsis cinerea*

*Coprinopsis cinerea* is an excellent model organism for studying the growth, development, and reproductive mechanism of other edible and medicinal fungi that are challenging to cultivate in laboratory conditions. With the completion of its whole-genome sequencing, it has been revealed to possess numerous crucial genes for synthesizing terpenoids and enzymes (Pei et al., 2018). Therefore, it is crucial to develop gene-editing technologies that are suitable for *C. cinerea*. In 2017, Sugano et al. (2017) constructed the CRISPR/Cas9 genome-editing system in *C. cinerea*, and it was optimized by a high-throughput transformation system. First, they developed a new method for protoplast preservation to reduce the preparation time of protoplasts. In addition, a novel stronger promoter-*CcDED*1_pro_ was screened to drive the Cas9 expression, and sgRNA was designed to target the *gfp* gene, which was constructed in the CRISPR/Cas9 vector and transformed into the protoplasts of a stable *gfp* expression line using the PEG-mediated method. Finally, the loss of *gfp* function was successfully detected. The cryopreserved protoplast is a powerful tool for high-throughput CRISPR/Cas9, thereby accelerating gene function studies and molecular breeding in edible fungi.

### 3.8. Applications of CRISPR/Cas9 genome-editing technology in *Schizophyllum commune*

*Schizophyllum commune* is a basidiomycetes fungus that provides a protein source of both high quality and quantity. However, the main challenge in gene deletion in *S. commune* is the relatively low incidence of homologous recombination, coupled with a relatively high incidence of NHEJ. In 2019, Jan Vonk et al. (2019) used pre-assembled Cas9 RNPs for high-throughput targeted gene deletion in *S. commune*. First, pre-assembled Cas9-sgRNA RNPs were used to delete the homeodomain transcription factor *hom2* in *S. commune*. However, it was found that Cas9 RNPs alone were not efficient enough for gene disruption in the absence of positive selection markers. Consequently, a repair template containing a selection marker was utilized in combination with Cas9-sgRNA RNPs to increase gene deletion rates. Sequencing analysis confirmed that the *hom2* gene was successfully replaced by the nourseothricin resistance cassette in all cases. Furthermore, an Δ*ku80* background was used to increase the rate of homologous recombination and reduce the number of ectopically integrated transformants, resulting in a higher gene deletion rate than in the wild-type strain. Finally, homology arms of 250 bp were identified to be sufficient for consistent recruitment of homology-directed repair. This report provides a new possibility for optimizing the CRISPR/Cas9 system to improve the genetic accessibility of non-model species.

### 3.9. Applications of CRISPR/Cas9 genome-editing technology in *Cordyceps militaris*

*Cordyceps militaris* is an *Ascomycete* species known for its bioactive constituents, such as cordycepin (Suhadolnik and Cory, 1964), cordyceps polysaccharide (Yu et al., 2004), and ergosterol (Bok et al., 1999). Studies have reported a diverse range of biological properties associated with these constituents, including anti-oxidant, anti-tumor, anti-aging, and anti-inflammatory (Das et al., 2010; Reis et al., 2013; Taofiq et al., 2016). It is meaningful to explore the synthetic mechanism of these substances and establish an efficient genome-editing method. However, there are still few reports on CRISPR/Cas9 genome editing in *C. militaris*, due to its complex genetic background and high proportion of fruiting body gemmated degeneration.

The first application of the CRISPR system in C. militaris was reported by Chen et al. (2018) in 2018, who successfully established an efficient CRISPR/Cas9 gene destruction system in the species. With the help of the newly discovered promoter *Pcmlsm3* and terminator *Tcmura3*, a vector containing three functional domains of the codon-optimized Cas9, sgRNA, and phosphinothricin acetyltransferase gene *blpR* was successfully constructed and transformed into wild-type *C. militaris* by PEG-mediated protoplast transformation. The stable expression of Cas9 was proved by fluorescent *gfp* tag and Western blot analyses. Regrettably, no *ura3* mutant was obtained, as the transcription level of sgRNA was extremely low. To circumvent the above problem, the strategy of *in vitro* introduction of sgRNA was adopted. The sgRNA and donor single-stranded DNA (ssDNA) synthesized *in vitro* were transformed into the *C. militaris* strains expressing *cmcas9* and screened by 5-FOA. The CRISPR/Cas9 system effectively generated mutants with site-specific deletions and insertions. As mentioned earlier, the application of the CRISPR system in the *C. militaris* is reported for the first time, which can greatly accelerate molecular breeding in *C. militaris*.

To develop powerful genome engineering tools, Chen et al. (2022a) successfully achieved multiplex gene precise editing and large DNA fragment deletion by the CRISPR/Cas9-TRAMA system in *C. militaris*. Leveraging the multiplex RNA-processing function of endogenous tRNA elements, the vector C9TRAMA was constructed by introducing the extrachromosomal plasmid and combining it with the homologous template of target sites. The marker-free CRISPR/Cas9-TRAMA multiplex gene-editing system, which can efficiently edit multiple targets at any position of the *C. militaris* genome to delete large synthetic clusters, was developed by transforming the vector into *C. militaris* via PEG-mediated protoplast transformation. Moreover, the greatest advantage of the Cas9-TRAMA system is that it could easily be removed without any accidental foreign DNA residue. The researchers constructed and transformed the vectors C9TRAMA-Cns1B, -Cns123, and-Egt1 to further edit the synthetases of cordycepin and ergothioneine, which proved that this system can be used for protein modification, promoter strength evaluation, and 10-kb metabolic synthetic cluster deletion. The Cas9-TRAMA system developed in this study provides a feasible approach for extracting valuable metabolic resources from medicinal mushrooms.

Because the AMA1 plasmid is difficult to insert into the fungal genome, it is easy to lose after being cultured under non-selective conditions, allowing for the reuse of dominant selection markers in the next transformation (Wang and Coleman, 2019). Therefore, in theory, the AMA1-based CRISPR/Cas9 genome-editing technology can achieve unlimited rounds of genetic engineering. In 2022, Meng et al. (2022) constructed an efficient CRISPR/Cas9 genome-editing system based on AMA1-based plasmids for the first time in *C. militaris*. The photoreceptor genes *Cmwc-1* and *Cmvvd* were selected as the target genes, as the appearance of albinism or deepened color of mycelium could confirm their disruption. The plasmids pAMA1-Cas9-sgRNA_Cmwc − 1_ and pAMA1-Cas9-sgRNA_Cmvvd_ were constructed and transformed into protoplasts by PEG-mediated transformation. The transformants were identified on a selective medium after light exposure, with efficiencies of 55.1 and 88.89%. To achieve a more precise target modification, mutants based on the vector harboring a sgRNA supplementation with an HDR template were obtained at an efficiency of 73.9%. Double-gene disruption of *Cmwc-1* and *Cmvvd* was also achieved with an efficiency of 10%. The CRISPR/Cas9 system with endogenous tRNA_Pro_ of *C. militaris* and the heterologous chimeric RNA Pol III promoter *AfU6-t RNA*_*Gly*_ exhibited an editing efficiency exceeding 80%, making them more suitable promoters. Finally, the transformation efficiency of PEG-mediated protoplast transformation and *Agrobacterium*-mediated transformation in *C. militaris* was compared. The results showed that the former had a notably higher efficiency than the latter. These findings hold tremendous potential for the utilization of CRISPR/Cas9 technology in characterizing gene function and molecular breeding of edible fungi in the future.

### 3.10. Applications of CRISPR/Cas9 genome-editing technology in *Shiraia bambusicola*

*Shiraia bambusicola*, a member of the Ascomycete family, thrives on the twigs of bamboo. Its fruiting bodies showcase a superior ability to produce high-value pharmacological drugs such as hypocrellin. This compound has been widely applied in many pharmaceutical fields, including the treatment of sciatica, pertussis, tracheitis, and rheumatic arthritis (Zhao et al., 2016). Unfortunately, inadequate molecular tools have impeded research and utilization of this promising compound. In 2017, Deng et al. (2017b) constructed the CRISPR/Cas9 system in *Shiraia sp*. SUPER-H168 and successfully deleted the key gene in the hypocrellin biosynthesis pathway-polyketide synthase (*SbaPKS*). First, the PlentiU plasmid carrying *hph* was used as the starting vector. The human *U6* promoter was used to activate the transcription of sgRNA, and the constitutive promoter *ef1*α was used to activate the expression of the Cas9 protein. The plasmids, harboring solely sgRNA and Cas9-*hph*, were transformed into the wild-type protoplast by PEG–CaCl_2_ transformation. There was no significant change in the morphology of the transformants compared with that of the wild-type strain, indicating that the Cas9 protein or sgRNA had no deleterious effect on the growth of the wild-type strain. Then, the PlentiU vector loaded with both sgRNA and Cas9-*hph* was transformed into *Shiraia sp*. SUPER-H168 to obtain the *SbaPKS*-edited mutant. The relative expression levels of *SbaPKS* and its adjacent genes in the Δ*SbaPKS* mutant were extremely downregulated compared with those of the wild-type strain, underscoring the involvement of *SbaPKS* in hypocrellin biosynthesis. The successful implementation of the CRISPR/Cas9 system in *S. bambusicola* will open a new window for deciphering the mechanism of hypocrellin biosynthesis and metabolites of medicinal fungi.

In 2020, Deng et al. (2020) constructed a more efficient CRISPR/Cas9 system in *S. bambusicola* for enhancing hypocrellin production by optimizing sgRNA transcription elements and interfering with the endogenous NHEJ pathway. 5SrRNA was selected for transcribing sgRNA, owing to the fact that the genome-editing efficiency of 5SrRNA CRISPR plasmid was higher than that of other *U6* mediating systems. In addition, the *ku80* gene was destroyed by the CRISPR system, and the homologous donor with *pyrG* cassette was integrated into the targeting genomic locus of the *ku80* gene via the DSBs repair system. The transformants were then screened on a regeneration medium containing 5-FOA. The results indicated that Δ*ku80* mutant was no damage to *S. bambusicola* and the homologous recombination efficiency was significantly increased to 100%. Subsequently, the optimized system was applied to improve hypocrellin production via gene modification and integration, increasing ~12-fold than that of the wild-type strain. This study lays the foundation for the regulation of metabolic pathways using the efficient CRISPR/Cas9 system in edible fungi.

## 4. Challenges and optimization of the CRISPR/Cas9 system in edible fungi

### 4.1. Low editing efficiency

Editing efficiency refers to the proportion of strains that achieve successful editing of target genes (Ruiping et al., 2019). Low editing efficiency is a common problem in the CRISPR/Cas9 system for edible fungi, which can be attributed to various reasons, including low transformation efficiency and regeneration efficiency of host material (Li et al., 2017), low expression level of Cas9 or sgRNA (Foster et al., 2018; Wang and Coleman, 2019), low targeting efficiency of sgRNA (Schuster et al., 2016; Xiao-tian et al., 2021) and low precision repair ratio (Yuan et al., 2017).

The optimization scheme can be considered from the following aspects: (1) Optimizing the genetic transformation system. It is necessary to establish stable and efficient genetic transformation systems (Li et al., 2017) given the complex cell wall structures of edible fungi. Combining multiple wall lytic enzymes under different digestion conditions may result in higher quality protoplasts (Liu X. et al., 2022). (2) Optimizing Cas9 expression and the sgRNA transcription strategy (Xuping et al., 2019). The insufficient expression of Cas9 is usually caused by the presence of rare codons. Therefore, codon optimization for target species is necessary (Liu et al., 2015; Ullah et al., 2020). Numerous facts prove that codon optimization makes the *Cas9* gene more efficiently expressed in specific species (Schuster et al., 2016; Qin et al., 2017; Chen et al., 2018; Wang et al., 2021; Liu X. et al., 2022). In addition, incorporating a suitable nuclear localization sequence to Cas9 protein is conducive to the specific expression of Cas9 protein in the nucleus of edible fungi, thereby improving the editing efficiency to a certain extent (Shi et al., 2019; Ullah et al., 2020). (3) The targeting efficiency of sgRNA affects the editing efficiency of CRISPR. Currently, there are many online tools to assist in the design of sgRNA for reference to select the optimal sgRNA. The related websites are shown in Table 2. (4) Selecting the appropriate promoter. Generally, endogenous *gpdA, TrpC*, and other promoters are selected to express *Cas9* gene, while the *U6* promoter is used to express sgRNA (Ruiping et al., 2019). (5) Improving repair accuracy. Some studies have shown that the efficiency of precise repair can be improved by inhibiting NHEJ repair or enhancing HDR repair (Yan and Finnigan, 2018; Tu et al., 2021).

**Table 2:** Part software tools of sgRNA design and off-target effect evaluation.

**Software brand**	**Website address**
CTISPRz	http://research.nhgri.nih.gov/CRISPRz/
GUIDE-Seq	https://www.illumina.com/science/sequencing-method-explorer/kits-and-arrays/guide-seq.html
sgRNA Scorer	https://crispr.med.harvard.edu/
CRISPR RGEN	http://www.rgenome.net/
E-CRISP	http://www.e-crisp.org/E-CRISP/
CHOPCHOP	http://chopchop.cbu.uib.no/
CCTop	http://crispr.cos.uni-heidelberg.de/
Cas-OFFinder	http://www.rgenome.net/cas-offinder/
CasOT	http://casot.cbi.pku.edu.cn/
CRISPR Design	http://crispr.mit.edu/
sgRNAcas9	http://www.biootools.com
CRISPRseek	http://www.bioconductor.org/packages/release/bioc/html/CRISPRseek.html
CRISPR-Cas	https://en.wikipedia.org/wiki/CRISPR
sgRNA Designer	http://www.broadinstitute.org/rnai/public/analysis-tools/sgrna-design
CRISPI	http://crispi.genouest.org/
WU-CRISPR	http://crispr.wustl.edu/
CRISPRdirect	http://crispr.dbcls.jp/
GT-Scan	https://gt-scan.csiro.au/
CRISPRscan	http://www.crisprscan.org/
CRISPRmap	http://rna.informatik.uni-freiburg.de/CRISPR-map/Input.jsp

### 4.2. Off-target effects

Off-target effects may exist in all species, and edible fungi are no exception. During the process of genome editing, base mismatches can occur between the sgRNA and the target DNA sequence (Fu et al., 2013) or sgRNA sequences, forming a DNA bulge that differs from the off-target DNA sequence (Lin et al., 2014) which can lead the sgRNA to pair with other bases. This can result in the cutting of non-target sequences and unnecessary mutations, which represents a major challenge of the CRISPR/Cas9 system. The off-target effect can be reduced via the following aspects: First, detecting the off-target effect in time. At present, many methods to detect the off-target effect have been developed. Chromatin immunoprecipitation sequencing (ChIP-seq) and genomewide, unbiased identification of DSBs enabled by sequencing (GUIDE-seq) are two such methods that could accurately identify off-target sites for CRISPR/Cas9 (Kuscu et al., 2014; Tsai et al., 2015; Lin et al., 2018; Rodriguez et al., 2021). Second, optimizing the sgRNA sequence. This can be achieved through the following steps: (1) Minimizing the number of base pairs between sgRNA and predicted off-target site sequences; sgRNA and predicted off-target sites should avoid four consecutive or interval base pairing; 1–5 bases near the PAM sequence have the greatest influence on the correct binding of sgRNA to the target DNA, so these five bases should be as specific as possible when designing sgRNA (Pei et al., 2018). (2) Modifying the length of sgRNA. Shortening the length of sgRNA from 20 nucleotides to 17–18 nucleotides (Fu et al., 2014) or adding two guanine nucleotides at the 5′ end of sgRNA (Cho et al., 2014) can significantly reduce undesired mutagenesis at some off-target sites. (3) Controlling sgRNA concentration. The off-target effect can be minimized by the reasonable concentration of the plasmid harboring sgRNA (Hsu et al., 2013; Pattanayak et al., 2013). Furthermore, using the RNA polymerase II transcription system to express sgRNA can better regulate the content of sgRNA, thereby minimizing off-target effects (Kiani et al., 2014).

Third, modifying the Cas9 protein. (1) Reducing or depriving the endonuclease activity of Cas9 protein. The Cas9 protein can be activated by non-targeting sequences within a certain mismatch range, resulting in mis-cleavage. To prevent this, mutants, such as H841A or D10A, which cleave the function of HNH or RuvC domains, can be used (Chiang et al., 2016). Alternatively, single-notch enzyme activity mutants, such as dCas9 and FokI-dCas9, can also be employed (Gao et al., 2016; Pan et al., 2016; Terao et al., 2016). These two methods can increase the degree of sequence matching required to activate Cas9 to a certain extent which significantly enhances the specificity of the system. (2) Reducing the ability of Cas9 protein to bind to DNA. Site-directed mutagenesis of Cas9 protein can be generated to form enhanced specificity Cas9 (eSpCas9) (Slaymaker et al., 2016) and high-fidelity-HF1 (SpCas9-HF1) (Kleinstiver et al., 2016). The mutants weakened the non-specific binding ability of Cas9 protein to DNA, thereby reducing off-target effects. (3) Enhancing the recognition ability of Cas9 protein to standard PAM sequence. This can be achieved by using Mutazyme II to randomly mutagenize the residues of SpCas9 and construct mutagenized PAM-interacting domain libraries. Positive selection and site-depletion assay of SpCas9 variants can then be performed to obtain variants with improved Cas9 PAM specificities (Kleinstiver et al., 2015). For example, the D1135E SpCas9 variant possesses improved discrimination against the off-targets with non-canonical NAG/NGA PAMs while retaining robust on-target activity on canonical NGG PAM sites (Kang et al., 2022). Fourth, controlling the action time of functional elements, such as Cas9 and sgRNA, in fungal cells. (1) Cas9 mRNA and sgRNA can be prepared by *in vitro* transcription and transferred into the cells (Nagy et al., 2017). Due to their susceptibility to degradation, the Cas9 and sgRNA will be eliminated rapidly, resulting in a shorter retention time of the CRISPR components in the cells. This approach can help reduce off-target effects. (2) The inducible promoter is used to express Cas9, so that Cas9 can only be expressed when the gene needs to be edited, such as alcohol dehydrogenase A (*alcA*) from *A.nidulans* (Waring et al., 1989) and benzoate p-hydrolase A (*bphA*) or catalase R (*catR*) from *Aspergillus niger* (Sharma et al., 2012; Antunes et al., 2016). (3) Using self-replicating plasmids. Cas9 in the plasmid will be lost from the cell during passage without screening pressure. (4) Deleting the functional elements, such as Cas9 and sgRNA, after editing by the CRISPR/Cas9 system using the FLP/FRT site-specific recombination system (Xuping et al., 2019), thus reducing the action time of CRISPR components in cells to reduce the off-target effect.

### 4.3. Difficulty in screening mutants

The difficulty in screening mutants in edible fungi arises from non-homozygous editing and fewer available selection markers (Deng et al., 2017a; Xiao-tian et al., 2021). Due to the heterokaryosis of edible fungi, many unedited nuclei in protoplasts are mixed in the screening of mutants, increasing the difficulty of screening. Therefore, many studies have utilized monokaryons of edible fungi for genome editing. It has been reported that the addition of chemical reagents, such as inositol and benomyl, increased the proportion of monokaryotic protoplasts in *Aspergillus oryzae*. This can greatly improve the obtaining efficiency of homozygous transformants and can avoid the multi-round single-spore isolation process in the late stage of transformation, thereby reducing the difficulty of screening (Zou et al., 2021). In addition, spore isolation to obtain mononuclear, haploid mutants, as the starting strain for gene editing, may be a solution (Han and Yixin, 2021). However, differences in morphological, physiological, and biochemical characteristics between monokaryons and dikaryons may need to be examined and circumvented.

Another limiting factor for mutant screening in edible fungi stems from the limited number of selective markers. Several commonly used markers have been used for screening positive transformants, including resistance genes (carboxin resistance gene and *hph*), auxotrophic markers (uridine auxotrophy), and mutagenic markers (*pyrG* and *ura3*). However, there are some limitations and potential problems with the above markers. For example, *hph* could not be stably expressed in *P. eryngii*. The transformants lost the *hph* in the third passage (Wang et al., 2021). The uracil auxotroph mutants can grow normally in a uridine-deleted medium only when the fragment containing the *ura3* expression element is correctly inserted (Chen et al., 2017). Recently, some scientists have proposed the design of recyclable cassettes that can be removed from the targeted genome after use for the reuse of selectable markers (Fuller et al., 2015). For example, Solis-Escalante et al. (2013) reported a novel recyclable dominant marker cassette *amdSYM* consisting of an *Ashbya gossypii TEF2* promoter and terminator and a codon-optimized acetamidase gene (*amdS*) from *A. nidulans* as the first dominant recyclable marker, which opens the door to rapid and simple genetic manipulation in industrial strains.

## 5. Future prospects of CRISPR/Cas9 system in edible fungi

### 5.1. Extending the PAM range

The PAM is located at the 3′-terminus of the target DNA, which is a short nucleotide motif of ~2–6 bp and is responsible for recognition by the Cas9 protein (Sternberg et al., 2014). Although the CRISPR/Cas9 genome-editing system has been widely used in edible fungi genome editing, its target selection is often restricted to DNA sequences with the NGG PAM motif, which severely hampers the scope of CRISPR/Cas9 genome editing in edible fungi. Researchers have been working to develop novel Cas9 variants to expand the scope of genome-targeted editing. SpCas9 variants, namely xCas9 and Cas9-NG, have been found to show great potential in improving targeting specificity and expanding targeting range. xCas9, for instance, can efficiently induce target site mutations of both NG and GAT PAM sequences (Hua et al., 2019). Cas9-NG can edit all NGG, NGA, NGT, and NGC sites, although with slightly reduced activity (Ren et al., 2019). Moreover, Cas9-NG can not only recognize NAC, NTG, NTT, and NCG apart from NG PAM (Ren et al., 2019) but can also achieve efficient editing at AT-rich PAM sites, such as GAT, GAA, and CAA (Zhong et al., 2019). Furthermore, researchers are actively exploring the discovery of new orthologous enzymes of Cas9 through sequence alignment, which is expected to significantly expand the application range of the CRISPR/Cas9 system. This development holds great promise for the genetic manipulation of edible fungi in the future.

### 5.2. Realization of multi-gene editing

Being a multicellular eukaryotic microorganism, edible fungi possess a more complex genetic background and tend to exhibit slower progress in genetic research as compared to bacteria and other prokaryotes. Consequently, most of the current reports on CRISPR/Cas9 in edible fungi mainly focus on the level of system construction and single gene editing (Song et al., 2019; Xuping et al., 2019). Traditional genetic approaches for continuous manipulating of multiple genes in edible fungi are neither efficient nor functional because of low homologous recombination rate and limited selection markers (Wang and Coleman, 2019). The CRISPR/Cas9 system could realize multiple gene editing by introducing multiple sgRNAs for different sites, or a single sgRNA for a conserved sequence of multiple sites at one time (Mali et al., 2013), which greatly facilitates study of the function of multiple genes (Xuping et al., 2019). Up to now, the following methods can be referred to achieve multiple gene editing: Multiple mature sgRNA synthesized *in vitro* could be introduced into Cas9-positive cells using the protoplast-mediated method. Co-transformation of sgRNA and donor DNA for different targets could realize multiple gene editing (Liu et al., 2015). In addition, the multiple RNA-processing functions of tRNA elements are expected to achieve multiple gene editing (Chen et al., 2022a). These methods are expected to provide a reference for multi-gene editing of edible fungi.

### 5.3. Application of base editors

In recent years, base editing technology based on CRISPR/Cas9 has gradually developed into a powerful genome-editing tool because of its advantages of no DSBs, no need for exogenous DNA templates, and no dependence on host homologous recombination repair. It has been developed and applied in animals (Komor et al., 2016; Kim et al., 2017; Zhang et al., 2017; Liu et al., 2018), plants (Shimatani et al., 2017; Zong et al., 2017; Tian et al., 2018), yeasts (Satomura et al., 2017; Pan et al., 2021), and bacteria (Zheng et al., 2018; Xia et al., 2020; Heo et al., 2022), which also provides a reference for the establishment and application of base editing systems in edible fungi. Base editors include cytosine base editor (CBE) and adenine base editor (ABE). CBE fuses catalytically impaired CRISPR/Cas9 mutant, cytidine deaminase enzyme, and uracil glycosylase inhibitor (UGI) to produce a base substitution from C-G to T-A near the sgRNA binding site (Komor et al., 2016). Similar to CBE, the difference is that ABE uses adenosine deaminase, which results in a base conversion from A-T to G-C (Gaudelli et al., 2017). As base conversion can be achieved without the introduction of DSBs, the base editor should be widely used for early termination of codon introduction and repair (Yannan and Yuhui, 2022). For instance, CBE can convert codons, such as CAA, CAG, and CGA to termination codons TAA, TAG, and TGA, respectively, thereby terminating gene expression prematurely. Similarly, ABE can convert the initiation codon ATG to ACG, which disrupts the initiation of gene translation. These base editors can be utilized for gene inactivation, investigating gene function, and directed modification of biological metabolic (Yannan and Yuhui, 2022). The establishment of base editors in edible fungi will greatly facilitate the study of gene function and the creation of high-quality germplasm resources.

## 6. Conclusion

**Figure 3:**
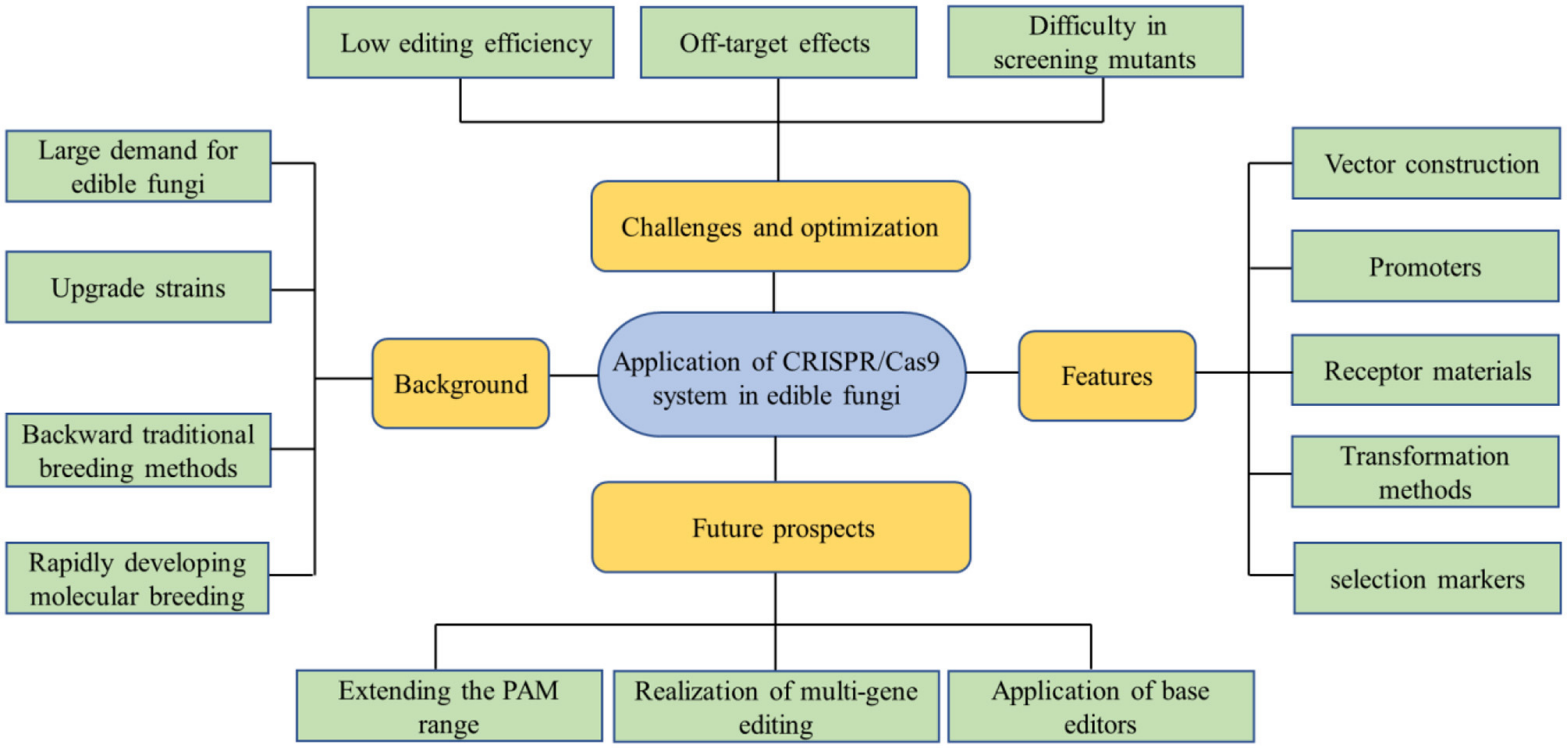
This concept map is used to summarize the entire article. Due to the intricate genetic background of edible fungi and the challenging nature of genetic manipulation, advancements in genetic research have been comparatively sluggish. However, with the swift progress of molecular biology, the CRISPR/Cas9 system is expected to have a significant impact on gene function research and molecular breeding of edible fungi.

The CRISPR/Cas9 genome-editing technology has already gained immense potential for a vast range of applications following its rapid development in recent years. Compared with ZFNs and TALENs systems, the CRISPR/Cas9 genome-editing technology system has shown great superiority with the advantages of simplicity, efficiency, and versatility. Meanwhile, it also shows irreplaceable application value in strain improvement, molecular breeding, and metabolite regulation of edible fungi. However, at present, there are still deficiencies in the application of this technology in edible fungi, such as off-target effect, low editing efficiency, and difficulty in mutant screening, which need more radical solutions. The advancement of the modern edible fungi industry is of great significance to ensure food safety, promote the development of ecological agriculture, and achieve the goal of “dual-carbon”. With the advent of the post-genome era and the in-depth utilization of the CRISPR/Cas9 technology, genome editing of edible fungi will step onto the right track, which will bring breakthroughs in gene mining of target traits, targeted improvement of varieties, and creation of new germplasm (Figure 3).

## Author contributions

QZ and LY designed the study and incorporated all the necessary modifications. YZ and SC drafted the manuscript and arranged the references. All authors have read and agreed to the published version of the manuscript.
